# Editorial: Transient ischemic attack: standard-of-care model

**DOI:** 10.3389/fneur.2023.1278624

**Published:** 2023-08-30

**Authors:** Narayanaswamy Venketasubramanian, Thanh G. Phan, Jiang Li, John Van Ly, Saeideh Aghayari Sheikh Neshin

**Affiliations:** ^1^Raffles Neuroscience Centre, Raffles Hospital, Singapore, Singapore; ^2^Department of Medicine, School of Clinical Sciences at Monash Health, Clayton, VIC, Australia; ^3^Department of Neurology, Monash Health, Clayton, VIC, Australia; ^4^Department of Molecular and Functional Genomics, Geisinger Medical Center, Danville, PA, United States; ^5^Neuroscience Research Center, Guilan University of Medical Sciences, Rasht, Iran

**Keywords:** transient ischemic attack, minor stroke, standard-of-care, large artery atherosclerosis, atrial fibrillation, sarcopenia

Approximately 20% of strokes, one of the leading causes of death and disability, are preceded by transient ischemic attacks (TIA). The definition of TIA has evolved from a mere symptom-based to a tissue-based definition using the presence of restrictive lesions in MR-DWI. Therefore, TIA is considered “a transient episode of neurological dysfunction caused by focal brain, spinal cord, or retinal ischemia, without acute infarction” (1). More recently, it has been argued that TIA might eventually be diagnosed as a minor ischemic stroke with improvement in neuroimaging. It was proposed to include TIA under a broader spectrum of acute cerebral ischemic syndrome. While TIA and minor strokes can at times be difficult to distinguish clinically, MRI of the brain may help if DWI restrictive lesions are detected. Despite the transient nature of TIA, it may herald subsequent devastating outcomes such as recurrent TIA, stroke, myocardial infarction, cognitive deficits, and death. Hence, there is an opportunity for prompt evaluation and appropriate early management to minimize the risk of adverse outcomes, especially as most recurrences occur in the first few weeks following the ictal event.

When evaluating a patient with suspected TIA, we should differentiate it from its non-ischemic mimics to decrease the rate of unnecessary hospitalization and to optimize healthcare utilization, but also to reduce the unnecessary risk of hemorrhage with escalation or long-term use of antithrombotics. A lack of consensus on the diagnosis of TIA among stroke specialists is (perhaps) due to no current standardized diagnostic criteria. In the acute setting, if available, cerebral CT perfusion and angiogram is important to rule out hemorrhage and to select the appropriate patients for reperfusion therapy. Once hemorrhage is ruled out and a critical decision regarding the need for reperfusion therapy has been made, the patient should be started on appropriate antiplatelet agent(s) and statin therapy. Based on the risk stratification of subsequent stroke, patients are often triaged for specialized inpatient or outpatient management, commonly with the use of ABCD2 score (2). However, the reliability and predictability of this score have been questioned in a meta-analysis (3). Around the world, there are various structured models of outpatient care of TIA patients available (4–6). The choice of the best model for caring for TIA patients depends on the local environment and healthcare resources. The care of these patients can be aided by the use of advanced neuroimaging and cardiac evaluations. It will remain to be seen if novel diagnostic and prognostic biomarkers can aid clinical decision-making.

In a previous meta-analysis of 18 randomized controlled trials (RCT) with acute non-cardioembolic TIA or IS (*n* = 15,515), compared to mono-antiplatelet therapy (MAPT), dual antiplatelet therapy (DAPT) reduced the risk of stroke recurrence (RR 0.69; 95% CI 0.61–0.78; *p* < 0.001) and composite vascular events (RR 0.72; 95% CI 0.64–0.80; *p* < 0.001) (7), but was associated with an increased risk of major bleeding (RR 1.77; 95% CI 1.09–2.87; *p* = 0.02). However, the benefit of DAPT in non-cardioembolic TIA or IS specifically due to large artery atherosclerosis (LAA) remains unclear.

In this Research Topic, Lin et al. performed a systemic review and meta-analysis by comparing DAPT vs. MAPT in patients with presumed symptomatic LAA. The authors included 10 trials (*n* = 5,004). Aspirin in combination with clopidogrel, cilostazol, or ticagrelor was compared to aspirin alone. Compared to MAPT, DAPT reduced IS recurrence (5.99 vs. 9.55%, RD: −3%, 95% CI: −5**–**0%). Intracerebral hemorrhage (ICH) and major bleeding were not significantly different: (0.28 vs. 0.32%, RD: 0%, 95% CI: −0**–**0%) and (0.73 vs. 0.51%, RD: 0%, 95% CI: −0**–**0%), respectively. However, the best DAPT regimen and treatment duration is still unclear. RCTs specifically investigating APT for LAA will be needed to provide evidence that will guide clinical practice.

Atrial fibrillation (AF) is an important cause of cardioembolic stroke. In a systematic review and meta-analysis of 17 studies (*n* = 1,163) of TIA, AF was detected in 4% (95% CI 2–7%) (8). The pick-up was higher among those who were older, had more extensive testing for arrhythmias, or were presumed cardioembolic/cryptogenic. While detection rates rose with a longer duration of monitoring, the included study with the longest follow-up was only 6 months.

Purroy et al. followed up 723 consecutive TIA patients for a median period of 6.5 (5.0–9.6) years. Using data from admission clinical assessments and investigations, as well as from follow-up review and medical records, the authors identified newly diagnosed AF (NDAF) in 116 (16.0%) patients: 42 (36.2%) during admission, 18 (15.5%) within the first year, 29 (25%) between one and five years, and 27 (23.3%) beyond 5 years. NDAF was associated with sex (female) (HR 1.61; 95% CI 1.07–2.41), age (HR 1.05; 95% CI 1.03–1.07), previous ischemic heart disease (HR 1.84; 95% CI 1.15–2.97), and cortical DWI pattern (HR 2.81; 95% CI 1.87–4.21). Patients with NDAF after admission and before 5 years of follow-up had the highest risk of subsequent ischemic stroke (log-rank test, P = 0.002). This study reinforces that careful patient selection (based on additional associated clinical and imaging criteria) could be a more cost-effective way of diagnosing NDAF.

Sarcopenia, characterized by loss of muscle strength and mass and reduced function, has been reported in 42% (95% CI 33%-52%) of 1,965 stroke patients included in seven studies in a systematic review and meta-analysis (9). However, the available number of studies on the effects of sarcopenia on stroke outcome had small sample size, short-term follow-up, or included patients undergoing rehabilitation, with no patients with TIA.

Lee et al. performed a retrospective study of the functional outcome as measured by modified Rankin score (mRS) at 90 days among 568 consecutive patients (mean age 65.5 ± 12.6 years, 64.6% male) with TIA or mild acute ischemic stroke (National Institute of Health Stroke Scale 0–5). Sarcopenia, defined as low muscle mass as measured by bioelectrical impedance analysis, or low muscle strength, was found in 48 (8.5%). It was associated with an unfavorable functional outcome defined as mRS 2-6 (OR 2.37; 95% CI 1.15–4.73) and an increase in the mRS score (OR 2.10; 95% CI 1.18–3.71). This study potentially suggests that sarcopenia may adversely affect stroke outcomes. A caveat is that this study did not include the baseline NIHSS as a covariate in the regression analysis. Further evaluation of sarcopenia is needed, including in stroke patients.

While it is thought that TIAs and minor stroke would have minimal impact on the patient after the acute event has passed, accumulated evidence suggests that there can be longer lasting fatigue, anxiety, depression, post-traumatic stress disorder, and cognitive impairment, as shown in a meta-analysis of 31 studies published in 2014 (10).

As the issues may evolve with time, Ebbesen et al. have proposed a protocol for a “living” meta-analysis that will collate the latest information on lasting impairments after TIA and minor stroke in the short- (< 3 months), medium- (3–12 months), and long-term (>12 months). Outcomes include fatigue, cognitive and communication impairments, depression, anxiety, quality of life, return to work/education, and social participation. Analysis will also be done separately for TIA and stroke. The study will provide useful and up-to-date information on these impairments and their impact, which will aid the healthcare team in their planning of patient-specific programs and, in a broader context, healthcare systems for these patients.

This Research Topic has brought forth useful new information that may impact the management of patients with TIA and minor stroke. This information also opens new grounds for fresh research that can improve the care of our patients.

## Author contributions

NV: Writing—original draft. TP: Writing—review and editing. JLi: Writing—review and editing. JVL: Writing—review and editing. SA: Writing—original draft.
